# Experiences of perinatal genetic screening for people from migrant and refugee backgrounds: a scoping review

**DOI:** 10.1038/s41431-024-01748-y

**Published:** 2025-01-04

**Authors:** Anaita Kanga-Parabia, Alison D. Archibald, Laura J. Biggs, Sharon Lewis, Erin Tutty, Belinda Dawson-McClaren

**Affiliations:** 1 Murdoch Children’s Research Institute, Melbourne, VIC Australia; 2 University of Melbourne, Melbourne, VIC Australia; 3 Victorian Clinical Genetics Services, Melbourne, VIC Australia

**Keywords:** Population screening, Genetic services, Population genetics, Patient education

## Abstract

People from refugee and migrant backgrounds often face poor experiences and outcomes in healthcare, and genetic healthcare is no exception. Understanding whether and how these health inequities manifest is an important step towards equitable perinatal genetic screening for genetic or chromosomal conditions (offered preconception, prenatally, or during the newborn period). A scoping review was conducted to review international evidence of perceptions and experiences of perinatal genetic screening for people from migrant and refugee backgrounds. Search strategies were applied to Medline, Embase, and CINAHL databases to identify articles meeting eligibility criteria. Evidence was synthesised using descriptive and content analysis, with theoretical frameworks of proportionate universality and relational autonomy used to interpret findings. Of 11,046 unique articles identified, twenty-six met inclusion criteria and underwent full-text review. Most studies were set in Western countries, and participants were primarily born in Asia, South America, or Africa. Studies indicated varying awareness, knowledge, attitudes, and uptake of screening. Several studies highlighted a lack of adequate in-language resources, the use of concepts that were unrecognised in particular communities, and poor interactions with healthcare providers. Strategies to address the above issues included person-centred counselling, increased consultation time, access to interpreters, and training for relevant providers. Other recommendations included addressing structural, financial, and geographical barriers to improve access to screening and associated care. Whilst additional research is required, we propose evidence and theory-informed strategies to improve perinatal genetic screening services for people from migrant and refugee backgrounds.

## Background

Perinatal genetic screening (‘screening’ herein) refers to screening with the primary purpose of identifying individuals and families with an increased chance of chromosomal and genetic conditions preconception, during pregnancy, or during the first year of life [1–3]. This includes reproductive genetic carrier screening offered before or in early pregnancy, aneuploidy screening for chromosome conditions offered during pregnancy, or newborn screening offered in early infancy [1–3]. Information from screening may help people to make informed reproductive decisions, and/or access early intervention and support for their children [1–3].

A key principle in delivering screening in most contexts is ensuring it is presented as optional and creating the opportunity for informed decision-making [4–6]. Informed decision making involves an individual making a deliberate decision that is aligned with their values and based on good knowledge about screening [7]. Informed decision making for screening may be facilitated by healthcare providers (HCPs) such as general practitioners, obstetricians, fertility specialists, and midwives, with the support of genetic HCPs such as genetic counsellors as required. However, HCPs involved in screening have cited a lack of time and complexity of genetic information as key barriers to discussing screening, particularly reproductive genetic carrier screening, and non-invasive prenatal testing [8, 9]. Digital tools such as online decision aids, portals, websites, factsheets, and chatbots have been used to supplement the above conversations, particularly as screening becomes more widely available [10–13]. It is important to understand whether this wide range of approaches is meeting the needs of the community. The first step in doing so involves understanding experiences and perceptions of screening. Specifically, this should include exploring experiences and perceptions of people who are known to face healthcare inequities, such as people from migrant and refugee backgrounds [14–17]. While definitions can differ widely across the literature, we use the term migrant to include people born outside the country in which they are residing, who have moved for various reasons including education or to be with family [18]. In contrast, people with a refugee background have been forcibly displaced from their country of nationality or usual residence. The term refugee background includes people who are legally deemed refugees as well as others with refugee-like experiences, including persecution, torture, trauma, loss of human rights, and separation from family members and friends [19]. Although important differences exist between migrant and refugee background experiences, these populations are infrequently and poorly delineated in the genetics literature. We use the term ‘people from migrant and refugee backgrounds’ to acknowledge the overlap and lack of consistency in terminology within the literature. Specific reference to ‘migrant’ or ‘refugee’ is used only when adequately defined by studies cited.

People from migrant and refugee backgrounds often have poorer healthcare experiences compared to those born locally [14–17]. This is particularly the case for people of refugee backgrounds, or those born in lower-income, conflict-affected, or non-Western countries [14–17]. Poorer experiences of healthcare may involve delayed or limited access to healthcare services, lack of information about available services, challenges in navigating the healthcare system, inadequate or incorrect health information, and lower empowerment or autonomy in relation to their healthcare [14–17]. Such experiences vary based on context and may be influenced by individual factors (e.g. English language proficiency), HCP factors (e.g. cultural sensitivity), and systemic factors (e.g. cost and location of healthcare services) [16].

While experiences are highly contextual, it is important to understand whether there are any common experiences and opportunities for intervention in the screening setting. An initial literature review revealed heterogeneity in the terminology used to describe both screening and participant demographics or identity. Articles that were identified described experiences of people from migrant and refugee backgrounds across several different screening and healthcare settings. Therefore, we conducted a scoping review, aiming to systematically identify and map global evidence regarding perceptions and experiences of screening for people of migrant and refugee backgrounds.

## Methods

Due to the broad exploratory nature of our research enquiry, we undertook a scoping review guided by the Joanna Briggs Institute approach [20] and the Preferred Reporting Items for Systematic Reviews and Meta-Analyses scoping review extension [21].

### Eligibility criteria

Eligibility criteria were determined through an iterative process as per the Joanna Briggs Institute approach, specifying population, context, concept, and source type (Table 1). In summary, papers were eligible for the scoping review if they were peer-reviewed original research articles reporting on uptake, experience, or perceptions of screening for people born outside the country in which the study was set.

**Table 1:** Eligibility criteria for scoping review, per Joanna Briggs Institute protocol [20].

	Inclusion criteria	Exclusion criteria
Population	Studies including people born outside the country in which the study was set. If the study population was mixed, studies were eligible only if country of birth was used as a comparator or if more than 90% of the sample were born overseas.	Studies in which country of birth was not clear
Context	Studies about screening in community, clinical, or research settings which aimed to identify increased chance of chromosomal and genetic conditions preconception, during pregnancy, or during the first year of life.	Studies about screening based on family history (such as genetic testing for Huntington’s disease), diagnostic testing (such as microarray on an amniocentesis sample) or testing for non-genetic health issues (such as cervical screening or mental health screening).
Concept	Studies exploring screening uptake, experience, or perceptions of screening.	Studies regarding detection rates, carrier frequency, and post-result experiences.
Source type	Peer reviewed original research articles.	Review articles, grey literature.

### Literature search

The search strategy was developed in collaboration with an experienced medical librarian. The search strategy was built iteratively, based on three constructs: people from migrant and refugee backgrounds; preconception and pregnancy genetic screening; and views and experiences. We included Medical Subject Headings (MeSH) terms, subject headings, and keywords. Once appropriate key search terms were selected, they were adapted to the requirements of three selected databases: Medline, Embase, and CINAHL. The primary search was undertaken on 22nd December 2022 and re-run on 2nd October 2024. The full search strategy is available in Supplementary Material 1.

### Study selection

Articles were imported to Zotero reference manager for removal of duplicates, then to Covidence systematic review software for screening [22]. All articles were screened by AK and ET in two stages: screening titles and abstracts; and screening full texts. Inconsistencies were resolved through discussion, involving others in the research team as required.

### Quality appraisal

Quality appraisal is not generally performed in a scoping review unless there is a specific reason to do so [20]. In this review, quality appraisal was not conducted. All studies meeting the selection criteria were included, with characteristics and quality of studies discussed where appropriate.

### Data charting and analysis

A data extraction form was created in Microsoft Excel, piloted by AK with five papers, and reviewed with the research team prior to use across all studies. Extracted data included details about source type (including year of publication and study type); participants (including country of residence and country of birth); context (including type of screening and study setting); and concept (including study aims and findings). Study characteristics were analysed using descriptive statistics. Total numbers varied due to overlapping categories, and/or missing data.

Results and discussion content were analysed using inductive content analysis [23]. This involved coding any content that related to the scoping review research question. Relevant content was initially coded into broad categories which were then compared and refined into more specific sub-categories and codes. Categories and codes were synthesised, interpreted and presented in a narrative format in the results section below. Data extraction and analysis were led by AK, with analyses refined and interpreted through discussions with the research team.

## Results

### Study selection

**Fig. 1:**
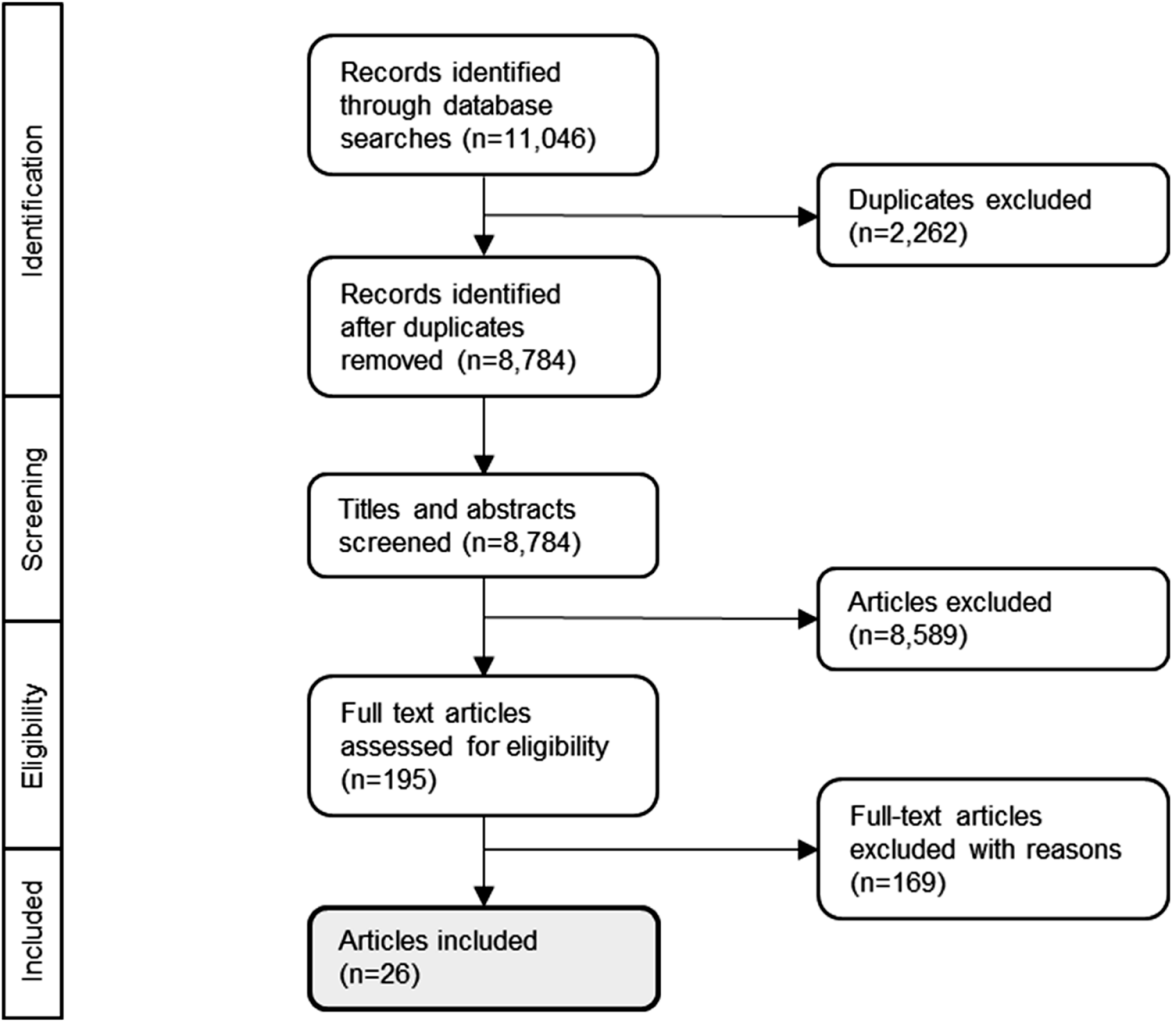
Scoping review process.

The literature search resulted in 11,046 records. After removing duplicates, 8,784 titles and abstracts were screened. One hundred and ninety-five full texts were screened, resulting in a total of 26 articles eligible for this scoping review (Fig. 1).

### Study characteristics

Study characteristics are described in Table 2. In summary, most studies included in the review: were conducted in the continents of Europe (11/26, 42%) and North America (9/26, 35%); included participants born in various regions (10/26, 38%) or focused on participants born in countries in Asia (9/26, 35%); reported on aneuploidy screening (18/26, 69%); explored experiences of screening offered in clinical settings outside the research study (19/26, 73%); and recruited participants from pregnancy care settings (15/26, 58%).

**Table 2:** Study characteristics.

Study characteristics	*N* (%)	Citations
Total articles included	26	[42, 24, 45, 46, 25–29, 43, 48, 30, 37, 26–29, 39–41, 31, 26–29, 39–41, 32–34, 38, 47, 49, 44, 35, 36]
Study setting (by continent)		
Europe	11 (42)	[42, 24, 45, 25, 26, 43, 41, 31, 32, 47, 49]
North America	9 (35)	[46, 28, 29, 48, 37, 33, 34, 38, 44]
Oceania	3 (12)	[30, 39, 40]
Asia	3 (12)	[27, 35, 36]
Participant region of birth (by continent)		
Various	10 (38)	[42, 24, 45, 25, 48, 30, 39, 41, 31, 33]
Asia^a^	9 (35)	[26, 27, 37, 40, 32, 38, 47, 35, 36]
South America	4 (15)	[28, 29, 34, 44]
Africa	3 (12)	[46, 43, 49]
Screening type^b^		
Aneuploidy screening	18(69)	[42, 24, 45, 46, 25, 28, 29, 43, 48, 37, 39–41, 31, 39–41, 32–34, 38]
Reproductive genetic carrier screening	8 (31)	[42, 26, 27, 30, 47, 49, 35, 36]
Genomic newborn screening	1 (4)	[44]
Screening context^c^		
Clinical setting external to study	19 (73)	[42, 24, 45, 25, 27–29, 43, 48, 30, 37, 39–41, 32–36]
Part of study	4 (15)	[28, 31, 47, 49]
Hypothetical	4 (15)	[46, 26, 38, 44]
Recruitment context		
Pregnancy care	15 (58)	[42, 24, 45, 25, 28, 43, 48, 39–41, 31–33, 44, 49]
General community	7 (27)	[46, 26, 27, 37, 38, 35, 36]
Genetics service	3 (12)	[29, 30, 34]
Preconception care	1 (4)	[47]
Study type		
Quantitative	13 (50)	[24, 45, 25, 27, 48, 39–41, 31, 33, 47, 49, 35]
Qualitative	11 (42)	[46, 26, 29, 43, 30, 37, 32, 34, 38, 44, 36]
Mixed methods	2 (8)	[28, 42]
Data source^d^		
Interviews	13 (50)	[42, 24, 46, 28, 29, 43, 30, 37, 32, 34, 38, 44, 36]
Surveys	11 (42)	[42, 24, 45, 25, 27, 28, 41, 31, 33, 47, 35]
Audits	5 (19)	[24, 48, 39, 40, 49]
Focus groups	3 (12)	[26, 34, 36]

^a^Turkey classified as Asia because majority of the country is in Asia and minority is in Europe.

^b^Total screening types exceed the total number of articles because one study reports experiences of multiple screening types.

^c^Total screening contexts exceed the total number of articles because one study reports on two screening contexts.

dTotal data sources exceed the total number of articles because some studies use multiple data sources.

Our synthesis revealed that the articles covered outcomes, experiences, perceptions and suggestions regarding [1]: screening awareness and information, and [2] screening attitudes, decisions, and access. Table 3 illustrates a summary of review findings which are further described below.

**Table 3:** Overview of scoping review synthesis showing two screening aspects for which outcomes, perceptions and experiences, and suggestions are reported by included studies.

	Screening awareness and information	Screening attitudes, decisions, and access
Outcomes	Participant awareness and knowledge	Participants attitudes toward screening Participant uptake of screening
Perceptions and experiences	Experiences receiving screening information Experiences of being offered screening	Participant beliefs and values about screening Involvement of healthcare providers in screening decisions Involvement of others in screening decisions Access to screening
Suggestions	Suggestions for screening information	Suggestions for decision support and access

### Screening awareness and information

Studies included in the review reported on participant awareness, knowledge, recall; experiences of receiving information and being offered screening; and suggestions for how information about screening should be delivered.

#### Participant awareness and knowledge

Thirteen studies reported on participant awareness and knowledge of screening [24–36]. Results from these studies are summarised below, with further details provided in columns K, L and M of Supplementary material 2. Most studies relied on participant self-reporting or subjective author assessments about awareness and knowledge of screening [24–30, 32, 34, 36]. Three studies calculated knowledge scores based on survey questions, which differed from study to study [28, 31, 33].

While outcomes varied within and across studies, over 50% demonstrated limited screening awareness and/or knowledge for participants born overseas (Table 4). There were no apparent trends in awareness and knowledge regarding study characteristics such as participant country of birth, study setting, or test type. However, one study found that higher knowledge scores were associated with English as a first language, a longer time in the country of residence, previous counselling about screening, and having more than one pregnancy [33]. This study reported no associations between knowledge and gestational age, maternal age, employment, marital status, education, income, ethnicity, and health literacy [33]. Overall, the studies provide evidence to suggest limited awareness and knowledge for people born overseas in several contexts. However, there are inconsistencies in awareness and knowledge assessment and variability in participant outcomes.

**Table 4:** Number of papers reporting on awareness and knowledge; attitudes; uptake of screening; and reasons for valuing or not valuing screening.

Study outcomes	*N* (%)	Citations
Awareness and knowledge	13	
Reported as adequate in most participants	4 (31)	[27, 29, 30, 34]
No association with country of birth	1 (8)	[33]
Negative association with overseas birth country	6 (46)	[24, 25, 28, 31, 35]
Limited awareness/ knowledge in most participants	2 (15)	[26, 32]
Attitude toward screening	8	
Reported as positive in most participants	4 (50)	[26, 30, 38, 44]
No association with country of birth	2 (25)	[45, 47]
Negative in most participants	1 (12)	[46]
Dependant on screening education method	1 (12)	[31]
Screening uptake	14	
Most participants had screening	6 (43)	[29, 48, 30, 40, 49, 35]
Positive association with overseas birth country	1 (7)	[48]
No association with country of birth	3 (21)	[25, 39, 47]
Negative association with overseas birth country	1 (7)	[24]
Most participants did not have screening	3 (21)	[28, 43, 32]
Reasons for valuing screening	11	
Information about pregnancy and/or child health	8 (73)	[42, 29, 30, 32, 34, 38, 44, 35]
Reassurance	6 (55)	[29, 43, 32, 34, 38, 44]
Preparing to have a child with a genetic condition	6 (55)	[42, 29, 30, 32, 38, 44]
Informing reproductive decisions	5 (45)	[42, 26, 43, 30, 32]
Perceived risk due to advanced maternal age	3 (27)	[29, 32, 34]
Perceived risk due to consanguinity	2 (18)	[27, 30]
To inform marriage partners	2 (18)	[26, 27]
Perceived risk due to family history of condition	1 (9)	[32]
Fulfilling sense of responsibility	1 (9)	[29]
Reasons for not valuing screening	7	
Feeling that results will not change anything	4 (57)	[46, 27, 30, 32]
Believing conditions predetermined by God	3 (43)	[30, 34, 38]
Believing conditions caused by environmental factors such as war, stress, infectious disease	2 (29)	[30, 36]

#### Experiences of receiving screening information

Those who were aware of screening had obtained information from several different sources, including non-genetics HCPs [25, 27, 32, 34, 36], genetics HCPs [28, 30, 34], family [28, 30, 34], friends [28, 34, 36], other community members [27, 30, 36], mass/ social media [27, 32], and marriage court [27]. However, studies also reported several issues relating to screening information for people born overseas: screening information was sometimes only available in languages they did not understand [29, 37, 32, 35, 36]; included concepts that are unrecognised in their community [25, 38]; lacked information on how to arrange testing [27]; and was generally confusing or inaccessible [25, 26, 32, 35]. In one study, several participants mentioned using a website providing general information for Korean immigrants in the US and identified a lack of screening information on this website [37]. Similarly, participants in another study mistakenly assumed that the information about screening provided via Japanese sources applied in Austria [32]. In some cases, participants also felt that their HCPs were uninformed about screening [26, 31].

#### Experiences of being offered screening

In addition to screening information sources, nine studies reported on the formal offer of screening when screening was offered in a clinical setting outside the study setting (Table 2). Two studies audited actual offer rates [39, 40], whereas other studies relied on participants self-reporting whether they were offered screening [24, 27, 28, 41, 31, 32, 34]. One study reported that country of birth was not associated with whether participants were offered screening [39]. However, other studies provided several examples of people born overseas not being offered screening or being offered screening too late for it to be relevant [24, 25, 27, 28, 40, 31, 32, 34]. In some cases, a lack of offer was due to late attendance for perinatal care [40, 41], or due to screening not being the most appropriate test for the individual [34]. Authors also speculated that low offer rates and understanding of screening may have been due to HCPs holding implicit biases, not having time, or lacking cultural competence [24, 25, 41, 31, 36].

#### Suggestions for screening information

Participants across several studies expressed a desire to receive information about screening and/or genetic risk, regardless of decisions they might make [42, 28, 29, 43, 41, 34, 44]. However, most suggestions about how the screening information should be delivered came from authors, often based on other existing literature or evidence generated in their study. Authors emphasized the importance of screening information being high-quality, timely, accurate, detailed, understandable, simple, clear, empathetic, and centralized [25–28, 30, 40, 32, 38, 44, 35, 36]. Some suggested that general public education may increase awareness for people born overseas [35, 36], whereas some highlighted the importance of targeted information aligning with community beliefs and practices [26, 28, 38, 35, 36].

Only four studies provided evidence of participant preferences for how screening information should be delivered [26, 30, 34, 36]. In these studies, participants emphasized the importance of screening information being high quality, timely, available in accessible locations outside the healthcare setting, delivered in multiple formats, available in multiple languages, and tailored for specific communities as required [26, 30, 34, 36]. For example, participants born in Pakistan and residing in the UK expressed a desire for public education, tailored for the Pakistani community with a belief that this may reduce stigma and enable individuals to discuss genetic risk more openly [26]. They emphasized a need to address disability, ‘options available for limiting disability’, as well as common areas of confusion in the Pakistani community, such as how consanguinity is related to genetic risk [26]. These results indicate limited evidence about participant preferences for screening information and demonstrate that both general and targeted approaches are required.

### Screening attitudes, decisions, and access

Studies included in the review described participant attitudes, uptake, beliefs, and values regarding screening. They also explored how social influences, healthcare provider involvement, and structural barriers influenced screening decisions and experiences.

#### Participants attitudes toward screening

Eight studies reported on participant attitudes toward screening, regardless of actual uptake [45, 46, 26, 30, 31, 38, 47, 44]. Results are summarised below, and further details are provided in N, O, and P of Supplementary material 2. Attitudes toward screening were based on pre-existing knowledge of screening [30, 45], screening information delivered during the study [46, 31, 47, 44], or hypothetical case studies [26, 38]. One study calculated an attitude score based on survey responses [31], and attitudes in the remaining studies were self-reported by participants in surveys or interviews [45, 46, 26, 30, 38, 47, 44]. Participants born overseas generally had a positive attitude towards screening, with variation within and between studies (Table 4). There were no apparent trends in attitudes toward screening regarding study characteristics such as participant country of birth, study setting, or test type. However, one study demonstrated that lower intention to have screening was associated with limited or absent national language proficiency, particular ethnicities, and living in less urban areas [41]. The study reported no association between maternal age or immigrant generation and intention to have screening [41]. Overall, these studies demonstrate variability within and between studies, with a skew toward positive attitudes about screening.

#### Participant uptake of screening

Fourteen studies reported on screening uptake [24, 25, 27–29, 43, 48, 30, 39–41, 32, 49, 35]. Results are summarised below, with further details in columns Q, R, and S of Supplementary material 2. Uptake was assessed in relation to screening offered either in a clinical setting external to the study [24, 25, 27–29, 43, 48, 30, 39–41, 32, 35], or as part of the research study [28, 49]. Screening uptake was either self-reported in surveys and interviews [24, 25, 27–29, 43, 30, 32, 35], based on clinical audit data [48, 39, 40, 49], or provided by HCPs [41]. There was variability in screening uptake across studies, with no apparent trends in uptake regarding study characteristics such as participant country of birth, study setting or test type (Table 4). However, one study found that uptake was associated with living in a non-urban area, receiving pregnancy care from a physician or midwife over an obstetrician, being younger, and being in a lower income quintile [48]. The study reported no association between screening uptake and maternal age during first pregnancy, history of pregnancy loss, history of congenital anomalies, or immigration status [48]. Overall, these studies demonstrated that screening uptake was primarily self-reported and varied within and between studies.

#### Participant beliefs and values about screening

Fourteen studies explored beliefs and values about screening of people born overseas. Those who saw value in screening did so for various reasons, with many believing that it would provide useful information about the health of their pregnancy or children (Table 4). Those who saw less value in screening felt the information would not change anything or believed that genetic conditions had non-genetic causes (Table 4).

Of note in the reviewed articles was a reported complex relationship between religion, spiritual beliefs, screening, and termination of pregnancy (TOP). Some participants felt that they would accept the child that God gave them and/or would not have a TOP because it was not allowed in their religion [42, 26, 30, 34, 38]. Others felt that they would terminate an affected pregnancy regardless of religious rules, or believed that TOP would be accepted by their religion in specific situations [42, 26, 43]. For some, screening felt meaningless if they would not consider further testing or TOP [46, 30, 32], whereas others saw value in screening even if TOP was not an option [42, 30, 34, 35].

Regardless of their decisions, some participants held concerns regarding screening [26, 27, 30, 44, 36]. Some concerns related to the testing process, such as accuracy [30, 44], privacy [30], time taken to test [30], and lack of trust in the result [27]. Other concerns related to implications of the diagnosis such as psychological impact of a diagnosis [30, 44]; whether they would receive adequate information and support [44]; how a diagnosis would be viewed by family, employees, or marriage prospects [26, 36]; and concerns about receiving incidental findings unrelated to the purpose of testing [30].

#### Involvement of healthcare providers in screening decisions

There were several examples of participants feeling that HCPs had empowered them to make their own decisions about screening [29, 32, 34]. However, some participants had testing because their HCP recommended it [27, 42], they believed it was compulsory [32], felt pressured to have screening [32], or it was mandated by a marriage court [27]. Some participants faced challenges in discussing their decisions with HCPs due to language barriers [37, 32, 36], rushed or ad hoc discussions [32], experiencing passivity when communicating with HCPs [32], concerns that HCPs would not share or understand their views [42], a sense that their culture was being criticised in relation to consanguinity [26], and previous experiences of discrimination perpetuated by HCPs [26, 36].

#### Involvement of others in screening decisions

Studies explored how participants involved or wished to involve other individuals in their screening decisions. The importance of including family in decisions about screening was highlighted in four studies with participants born in Southeast Asia, East Asia, and Latin America [42, 29, 32, 38]. Involvement of partners varied, with some not involving their partners in screening decisions, some wishing to involve their partner in decisions, and some prioritising their partner’s perspective [42, 29, 43, 32]. In one study, participants expressed frustration about a lack of in-depth communication and support from their partner [32]. Participants in another study tended to seek support from their partner or family as needed and saw HCPs as an information source only [38].

Some studies also commented on the influence of individuals who were not directly involved in decisions. Participants in three studies were influenced by the screening experiences of their family and friends [29, 32, 36]. In a study conducted with people born in Somalia residing in USA, participants were not supportive of screening because TOP was not accepted by their community [46]. Conversely, in a study conducted with people born in Southeast and East Asia residing in USA, participants raised that people may feel social or family pressure to have screening and/or terminate pregnancies due to lack of resources available for people with disabilities in Asian countries, a fear of shame or negative judgement, or lack of exposure to disability [38]. Some participants worried about being judged as irresponsible if they chose not to have screening [42], whereas others commented that they were not concerned about other people’s opinions regarding their screening decisions [30, 42].

#### Access to screening

Ten studies reported on structural facilitators and barriers that could impact access to information, support, and screening itself [42, 45, 27, 29, 43, 37, 32, 38, 35, 36]. In the context of funded screening, participants appreciated that it was free of charge [42]. Participants in two studies also commented that screening was more available in the country of residence compared to their country of origin [29, 32]. However, some faced barriers such as cost of screening and associated care [45, 27, 29, 43, 37, 32, 38, 35, 36]; inconvenient locations or processes [29, 37, 35, 36]; reluctance to seek healthcare due to fear of deportation [29, 36]; and lack of resources to meet specific needs of people born overseas [36].

#### Suggestions for decision support and access

Six studies explored participant expectations for HCP involvement in screening decisions. Some participants valued individual autonomy and saw HCPs as a source of information or emotional support [42, 29, 43, 38, 44]. Conversely, some wanted directive advice from HCPs about whether to have screening [42, 38, 44, 36], felt screening should be compulsory [42], or felt it would be challenging to say ‘no’ if an HCP offered it [42]. Reasons for these views included a lack of confidence to make screening decisions on their own or believing that not everyone would be able to decide for themselves. Others felt that their HCP may hold fatalistic beliefs and would therefore recommend against screening [38]. There were no apparent commonalities in these studies in relation to participant country of birth, study setting or test type. Only one study provided participant suggestions of how to address decision support needs, and most participants in this study worked in healthcare themselves [38]. Participants in this study suggested that HCPs disclosing what they would do in a similar situation could facilitate rapport, encourage people from Asian backgrounds to engage in the decision-making process, and remove the individual’s potential burden of making a decision without providing explicit direction [38].

Additionally, two studies explored the approach of discussing screening in a group prenatal care setting, with authors highlighting that this may be more efficient and cost-effective than individual counselling [29, 34]. Some participants saw the group approach as an opportunity for social support and to learn from others in similar situations, particularly when living away from family [29, 34]. Others expressed a preference for individual appointments to ensure privacy and a focus on their individual circumstances [29, 34].

The studies provided limited evidence of participant preferences and needs regarding decision support and no participant suggestions regarding structural access to screening. However, several authors made their own suggestions. Most of these authors highlighted the importance of person-centred counselling through suggestions such as recognising diversity within groups, considering cultural beliefs and practices when discussing screening, incorporating family dynamics into screening decisions, assessing individual expectations of the HCP role in decision making, and using written or visual aids as required [42, 46, 26, 28, 29, 43, 38]. Authors also conveyed that HCPs need to be provided with resources such as time, and access to interpreters for adequate discussions about screening to occur [25, 28–30, 40, 36].

Authors in some settings suggested a need for education informing HCPs about relevant cultural beliefs, practices, and implicit biases, as well as screening education for other staff such as social workers and interpreters [24, 46, 25, 43, 36]. Structural access issues were considered from the context of carrier screening in Thailand [35, 36]. Authors suggested approaches such as a government-funded thalassaemia prevention programme for migrants, which already exists for citizens [35, 36]. They also suggested offering screening in more convenient locations to address participant concerns such as missing work and losing income to attend hospital care [35, 36]. However, it was unclear as to where the ideal location would be, due to issues such as screening in workplaces requiring employer engagement and raising confidentiality concerns, or screening in schools or premarital settings relying on people migrating in time to be offered [35, 36].

## Discussion

This is the first review of international evidence regarding perinatal genetic screening for people from migrant and refugee backgrounds. The review highlighted the variation in experiences, views, and preferences across individuals and communities, as well as potential areas for improvement for screening services. Variability in experiences and views was apparent across different healthcare contexts. Furthermore, it was clear that other factors such as language, religion, and family dynamics influenced experiences. However, our understanding of the intersectionality of these factors was limited by what was reported.

Proportionate universalism can help to explain and contextualise many of the issues related to screening awareness and information raised by this review. Proportionate universalism advocates for the delivery of universal services at a scale and intensity proportionate to level of need [50]. This approach aims to improve health across the whole population while recognizing that needs fall on a continuum [50]. Taking a completely universal or standard approach to delivering healthcare services may disproportionately advantage those who are most able to access the services. Thus, targeted approaches are necessary to achieve health equity [50, 51].

In the context of screening, issues such as receiving inadequate information, not being offered screening in time for it to be useful, or facing structural barriers have also been identified at the broader population level [52–54]. Therefore, strategies such as educating HCPs about screening, raising public awareness, and providing access to information about screening in different formats may universally improve screening experiences.

The review also identified considerations about the above issues as well as novel issues that are unique to people of migrant and refugee backgrounds. This included lack of in-language information and support, information that does not consider cultural beliefs and expectations, and HCP implicit biases. Therefore, targeted approaches for people of migrant and refugee backgrounds may include improving language access, providing HCPs with education about cultural safety, considering community specific beliefs or concerns about disability and screening, and addressing structural barriers. Specific interventions may be context-dependent; therefore, health equity and language access frameworks, such as the ‘Cultural responsiveness framework - guidelines for Victorian health services’ [55], recommend that needs assessments should be conducted in any context where this has not previously been explored.

With regard to screening attitudes and decisions, motivations and concerns about screening that were identified in these studies primarily reflected those in broader population studies [56–58]. For example, seeking information to inform reproductive decisions or access early diagnosis and treatment are common reasons to pursue screening [56, 57, 59]. These findings emphasize the importance of supporting individuals, including people from migrant and refugee backgrounds, to make screening decisions considering their own beliefs and values.

Notably, several participants experienced, expected, or desired more involvement or direction from their HCP, partner, or other family members. However, informed decision-making is often framed as an autonomous decision being made in relation to the individual’s beliefs with minimal involvement from others [60]. While minimising involvement from others aims to protect individuals from coercion, pressure, or excessive influence, it has also been challenged as being too narrow, placing too much emphasis on the information aspect of informed decision-making, and not always considering the social contexts within which people make health and screening decisions [61–65]. This may particularly be the case for people from more collectivist cultures given close connections between informed decision-making, autonomy, and Western ideologies of individualism [66, 67].

An alternative view of autonomy, relational autonomy, acknowledges that others play a central role in decision-making [60]. Relational autonomy encourages HCPs to respect the individual while also encouraging them to consider their social situation and influences [60]. Suggestions made by the authors of studies included in this review may assist HCP to take a more relational approach to facilitating informed decision-making, including practicing person-centred care, considering family dynamics, and assessing what the individual expects from the HCP. However, these suggestions primarily come from authors rather than participants themselves. Further research from the perspective of community and HCPs is required to further understand how to best meet the needs of people from migrant and refugee backgrounds in healthcare interactions regarding screening.

Using evidence mapped in this review, and connecting to the theoretical concepts discussed, we propose several strategies to improve screening services for people from migrant and refugee backgrounds (Table 5). These strategies may be applied at the healthcare system and/or service level, or by individual healthcare providers and may be aimed at improving screening experiences universally or targeting groups or individuals who experience inequities.

**Table 5:** The way forward: evidence-based and theory-informed strategies to improve perinatal genetic screening services for people from migrant and refugee backgrounds.

	Healthcare system and/or services providing screening	Healthcare provider
Universal improvement of screening services	• Address structural barriers to accessing screening such as cost and location at a universal level. • Provide high quality, timely information about screening in multiple formats. • Make screening information available outside traditional healthcare settings.	• Offer screening consistently, without assumption of desire for screening. • Offer screening early. • Take a relational approach to screening discussions. • Consider that there is diversity within groups. • Use written or visual aids.
Targeted approaches for people from migrant and refugee backgrounds	• Address structural barriers to healthcare access such as cost and location for specific communities. • Allocate resources to support use of interpreters, longer appointments, healthcare provider training, etc. • Conduct research to develop targeted interventions for specific migrant and refugee communities. • Tailor screening information for specific communities. • Provide screening information in multiple languages. • Provide screening education for interpreters	• Consider cultural beliefs and practices in discussions about screening decision-making. • Learn about cultural safety and working with interpreters.

### Limitations

Retrieving and mapping evidence in this scoping review was limited by several factors. Heterogeneity of terminology and definitions of terms within the literature may mean that we have not captured all papers relevant to the review. For example, several articles were excluded due to a lack of clarity about whether the study was about screening or diagnostic testing. Articles were also excluded if it was not clear as to whether the study was describing country of birth, ethnicity, or race.

While there are several characteristics and concepts that have a close relation to country of birth, such as language, nationality, ethnicity, and race, we limited the eligibility criteria to be based on the objective construct: country of birth relative to country of residence/study conduct. Concepts such as language, nationality, ethnicity, and race may be included in the eligibility criteria of future reviews to further develop our understanding of how communities experience screening.

Furthermore, while the findings of the included studies are highly contextual, the breadth of evidence highlights key considerations for delivering screening programmes for people of migrant and refugee backgrounds.

### Conclusion

This scoping review aimed to map global evidence of perceptions and experiences of perinatal genetic screening for people from migrant and refugee backgrounds. Similar to the studies within the broader population, studies with people from migrant and refugee backgrounds highlighted varying screening awareness, knowledge, attitudes, beliefs, and uptake. Some issues experienced by people from migrant and refugee backgrounds have also been identified in research about the broader population. However, people from migrant and refugee backgrounds experience unique issues such as a lack of screening information in their language, information not tailored to their culture or context, and interactions with healthcare providers that did not meet their expectations or needs. There is a clear need for approaches that are targeted and contextual to the needs of specific communities from migrant and refugee backgrounds. This scoping review provides overarching considerations for health systems, screening services, and HCPs to work toward equitable perinatal genetic screening. Further research could support the development of interventions for specific migrant and refugee communities, and an understanding of what types of support are needed to enable HCPs and systems to deliver more culturally sensitive screening services.

## Supplementary information

Search strategy

Summary of article findings
