# Supplementary material for: Experiences of perinatal genetic screening for people from migrant and refugee backgrounds: a scoping review
Source: Eur J Hum Genet. 2025 Jan 4;33(6):701–10. doi: 10.1038/s41431-024-01748-y (PMC12185760; doi:10.1038/s41431-024-01748-y)
Supplement: Supplementary file 1 — Search strategy [file 41431_2024_1748_MOESM1_ESM.docx]

# Supplementary material 1 - search strategies

*RE: Kanga-Parabia et al. Experiences of perinatal genetic screening for people from migrant and refugee backgrounds: a scoping review, European Journal of Human Genetics (2024)*

Medline search conducted 21/12/2022 and 2/10/2024

| 1. | "Transients and Migrants"/ or exp "emigrants and immigrants"/ or refugees/ or minority groups/ or "Emigration and Immigration"/ or exp Ethnic Groups/ or culturally competent care/ or Cultural Competency/ or cultural characteristics/ or cultural diversity/ |
| --- | --- |
| 2. | (ethnic* or asylum-seek* or refugee* or emigrant* or emmigrant* or imigrant* or immigrant* or emigration* or emmigration* or imigration* or immigration* or migrant*).tw,kf. |
| 3. | (non-english-speaking or limited-english-proficiency or LOTE or CALD or LEP or NESB).tw,kf. |
| 4. | ((language or linguistic* or communication or low) adj3 (barrier* or proficien* or english or divers* or difficult*)).tw,kf,hw. |
| 5. | "Ethnic and Racial Minorities"/ or racial groups/ or racial groups/ or blacks/ or american native continental ancestry group/ or asians/ or whites/ or "native hawaiian or other pacific islander"/ or ethnicity/ or "hispanic or latino"/ |
| 6. | 1 or 2 or 3 or 4 or 5 |
| 7. | *Genetic carrier screening/ |
| 8. | Noninvasive Prenatal Testing/ |
| 9. | (Heterozygote-detection or carrier-screen*).tw,kf. |
| 10. | ((Genetic or aneuploid* or chromosom* or prenatal or antenatal or ante-natal) adj3 (screen* or test*)).tw,kf. |
| 11. | (NIPT or NIPS or cell-free-DNA-test*).tw,kf. |
| 12. | Neonatal Screening/ |
| 13. | ((infant or newborn* or neonat*) adj3 screen*).tw,kf,hw. |
| 14. | 7 or 8 or 9 or 10 or 11 or 12 or 13 |
| 15. | (Uptake or intake or Participat* or Access* or Utilisation or Utilization or Experience* or Barrier* or Facilitat* or Support* or Opinion* or View? or Offer* or Perception* or Perceive*).tw,kf. |
| 16. | Health Knowledge, Attitudes, Practice/ |
| 17. | "delivery of health care"/ or culturally competent care/ or health services accessibility/ or healthcare disparities/ or Cultural Competency/ |
| 18. | 15 or 16 or 17 |
| 19. | 6 and 14 and 18 |
| 20. | limit 19 to (case reports or comment or editorial or guideline or letter or practice guideline or preprint) |
| 21. | 19 not 20 |

Embase search conducted 21/12/2022 and 2/10/2024

| 1. | exp migrant/ or exp refugee/ or minority group/ or exp migration/ or exp ethnic group/ or transcultural care/ or cultural competence/ or cultural factor/ or cultural diversity/ |
| --- | --- |
| 2. | (ethnic* or asylum or refugee* or emigrant* or emmigrant* or imigrant* or immigrant* or emigration* or emmigration* or imigration* or immigration* or migrant*).tw,kf,dq. |
| 3. | (non-english-speaking or limited-english-proficiency or LOTE or CALD or LEP or NESB).tw,kf,dq. |
| 4. | ((language or linguistic* or communication or low) adj3 (barrier* or proficien* or english or divers* or difficult*)).tw,kf,hw,dq. |
| 5. | ethnicity/ or exp ancestry group/ or exp black person/ or american indian/ or exp asian/ or exp caucasian/ or exp oceanic ancestry group/ or exp hispanic/ |
| 6. | 1 or 2 or 3 or 4 or 5 |
| 7. | heterozygote detection/ |
| 8. | noninvasive prenatal testing/ |
| 9. | newborn screening/ |
| 10. | (Heterozygote-detection or carrier-screen*).tw,kf,dq. |
| 11. | ((Genetic or aneuploid* or chromosom* or prenatal or antenatal or ante-natal) adj3 (screen* or test*)).tw,kf,dq. |
| 12. | (NIPT or NIPS or cell-free-DNA-test*).tw,kf,dq. |
| 13. | ((infant or newborn* or neonat*) adj3 screen*).tw,kf,hw,dq. |
| 14. | 7 or 8 or 9 or 10 or 11 or 12 or 13 |
| 15. | (Uptake or intake or Participat* or Access* or Utilisation or Utilization or Experience* or Barrier* or Facilitat* or Support* or Opinion* or View? or Offer* or Perception* or Perceive*).tw,kf,dq. |
| 16. | attitude to health/ |
| 17. | health care delivery/ or exp health care access/ or exp transcultural care/ or health care disparity/ or cultural competence/ |
| 18. | 15 or 16 or 17 |
| 19. | 6 and 14 and 18 |
| 20. | limit 19 to (editorial or letter or note or "preprint (unpublished, non-peer reviewed)") |
| 21. | 19 not 20 |

PubMed search conducted 21/12/2022 and 2/10/2024

| 1. | title/abstract  “ethnic*” OR “asylum-seek*” OR “refugee*” OR “emigrant*” OR “emmigrant*” OR “imigrant*” OR “immigrant*” OR “emigration*” OR “emmigration*” OR “imigration*” OR “immigration*” OR “migrant*” OR “minority-group*” OR “cultural-competen*” OR “cultural-divers*” OR “racial*” OR “black*” OR “american-native*” OR “american-indian*” OR “asian*” OR “white*” OR “caucasian*” OR “native-hawaiian*” OR “oceanic-ancestry*” OR “pacific-islander*” OR “Hispanic*” OR “latino*” OR “transcultural-care*” OR “cultural-factor*” OR “limited-english-proficiency” OR “LOTE” OR “CALD” OR “LEP” OR “NESB” OR “non-english-speaking” |
| --- | --- |
| 2. | title/abstract  (“language” OR “linguistic*” OR “communication” OR “low”) AND (“barrier*” OR “proficien*” OR “english” OR “divers*” OR “difficult*”) |
| 3. | #1 OR #2 |
| 4. | title/abstract  “Heterozygote-detection” OR “NIPT” OR “NIPS” OR “cell-free-DNA-test*” |
| 5. | title/ abstract  (“Genetic” OR “carrier” OR “aneuploid*” OR “chromosom*” OR “prenatal” OR “antenatal” OR “ante-natal” OR “infant” OR “newborn*” OR “neonat*”) AND (“screen*” OR “test*”) |
| 6. | #4 OR #5 |
| 7. | title/ abstract  “Uptake” OR “intake” OR “Access*” OR “Utilisation” OR “Utilization” OR “Experience*” OR “Barrier*” OR “Facilitat*” OR “Support*” OR “Opinion*” OR “View” OR “Views” OR “Offer*” OR “Perception*” OR “Perceive*” OR “attitude*” OR “cultural-competen*” OR “delivery-of-health*” OR “healthcare-deliver* health-service-access*” OR “healthcare-disparit*” |
| 8. | all fields  NOTNLM OR publisher[sb] OR inprocess[sb] OR pubmednotmedline[sb] OR indatareview[sb] OR pubstatusaheadofprint |
| 9. | #3 AND #6 AND #7 AND #8 |
| 10. | Excluded  (“case reports” OR “comment” OR “editorial” OR “guideline” OR “letter” OR “practice guideline” OR “preprint”) |
| 11. | Excluded  (GeneReviews[books]) |
